# Automated Ecological Assessment of Physical Activity: Advancing Direct Observation

**DOI:** 10.3390/ijerph14121487

**Published:** 2017-12-01

**Authors:** Jordan A. Carlson, Bo Liu, James F. Sallis, Jacqueline Kerr, J. Aaron Hipp, Vincent S. Staggs, Amy Papa, Kelsey Dean, Nuno M. Vasconcelos

**Affiliations:** 1 Department of Pediatrics, Children’s Mercy Kansas City, Kansas City, MO 64108, USA; vstaggs@cmh.edu (V.S.S.); aepapa@cmh.edu (A.P.); kmdean@cmh.edu (K.D.); 2 Department of Electrical and Computer Engineering, University of California San Diego, La Jolla, CA 92093, USA; boliu@eng.ucsd.edu (B.L.); nuno@ece.ucsd.edu (N.M.V.); 3 Department of Family Medicine and Public Health, University of California San Diego, La Jolla, CA 92093, USA; jsallis@ucsd.edu (J.F.S.); jkerr@ucsd.edu (J.K.); 4 Department of Parks, Recreation and Tourism Management, North Carolina State University, Raleigh, NC 27695, USA; jahipp@ncsu.edu

**Keywords:** accelerometry, exercise, measurement, parks, public health

## Abstract

Technological advances provide opportunities for automating direct observations of physical activity, which allow for continuous monitoring and feedback. This pilot study evaluated the initial validity of computer vision algorithms for ecological assessment of physical activity. The sample comprised 6630 seconds per camera (three cameras in total) of video capturing up to nine participants engaged in sitting, standing, walking, and jogging in an open outdoor space while wearing accelerometers. Computer vision algorithms were developed to assess the number and proportion of people in sedentary, light, moderate, and vigorous activity, and group-based metabolic equivalents of tasks (MET)-minutes. Means and standard deviations (SD) of bias/difference values, and intraclass correlation coefficients (ICC) assessed the criterion validity compared to accelerometry separately for each camera. The number and proportion of participants sedentary and in moderate-to-vigorous physical activity (MVPA) had small biases (within 20% of the criterion mean) and the ICCs were excellent (0.82–0.98). Total MET-minutes were slightly underestimated by 9.3–17.1% and the ICCs were good (0.68–0.79). The standard deviations of the bias estimates were moderate-to-large relative to the means. The computer vision algorithms appeared to have acceptable sample-level validity (i.e., across a sample of time intervals) and are promising for automated ecological assessment of activity in open outdoor settings, but further development and testing is needed before such tools can be used in a diverse range of settings.

## 1. Introduction

While person-worn devices are commonly used to assess physical activity of individuals, ecological assessment, primarily direct observation, is often used to assess physical activity within specific settings (e.g., parks, schools) [1]. Ecological assessment of physical activity, defined here as the assessment of aggregated (across people) amounts of physical activity within settings, is distinct from individual-level assessment in that the former provides physical activity metrics for a setting, rather than an individual.

Ecological direct observation measures have a long history of use for assessing both the volume (e.g., minutes of physical activity) and distribution (e.g., proportion of people physically active) of activity in various settings to gauge the relative contribution of specific settings to physical activity, identify environmental influences of physical activity, and evaluate setting-based physical activity interventions [1,2]. Among the most commonly used ecological direct observation measures are those for parks (e.g., System for Observing Play and Recreation in Communities; SOPARC) [3] and schoolyards or gymnasiums (e.g., System for Observing Fitness Instruction Time; SOFIT) [4]. These tools have been used to investigate the impact of park renovations on park use and park-based physical activity [5], conduct national surveillance of physical activity in parks [6], and evaluate and improve physical activity in physical education [7].

Limitations of existing direct observation tools for ecological assessment of physical activity include that they are not feasible for providing continuous monitoring and rapid feedback to prompt improvements in environments and programs, only capture each individual in the setting for a brief moment, and require significant resources, such as training observers, and conducting time-consuming manual observations. Thus, observations are often for a limited time period that may not be fully representative. Further, data processing is time consuming and data visualization is lacking.

Computer vision is an engineering approach that involves processing visual information from digital cameras using automated algorithms and has promise for advancing the ecological assessment of physical activity. For example, automated processing systems could facilitate the continuous ecological assessment of physical activity in settings with existing (e.g., security) or purposefully-placed video cameras to provide rapid feedback to inform environmental and/or programming interventions. Investigating such technologies has been identified as a top priority in physical activity observation research [2].

Only one previous study investigating this type of technology could be located, and findings were promising [8]. However, two aspects of the previous study limited the technology’s ability to be used in diverse outdoor settings, such as parks and schoolyards. First, only one indoor gymnasium was captured, with little-to-no variability in lighting, and the camera was affixed directly overhead to eliminate occlusions, both of which are not practical in most outdoor environments. Second, the algorithm used vest colors to track participants, meaning each participant had to wear a special-colored vest, which is not feasible in most public settings (e.g., parks) [9]. The computer vision approach used in the present study was designed to perform outdoors and have the ability to adapt to different views and scenes, making it more likely to generalize across diverse settings.

The present proof-of-concept study developed and evaluated criterion validity of novel computer vision algorithms for ecological assessment of both volume and distribution of physical activity in one open park setting. Person-worn accelerometers were used as the ground truth (criterion) measure for training and testing the algorithm. Accelerometers were selected because they provide high-frequency data (e.g., every second), whereas existing direct observation tools, such as SOPARC, do not provide high-frequency data (e.g., one moment every 15 min). High-frequency data are necessary for training movement-based algorithms and allow for a larger sample size for validity testing.

## 2. Materials and Methods

### 2.1. Participants and Procedures

**Figure 1:**
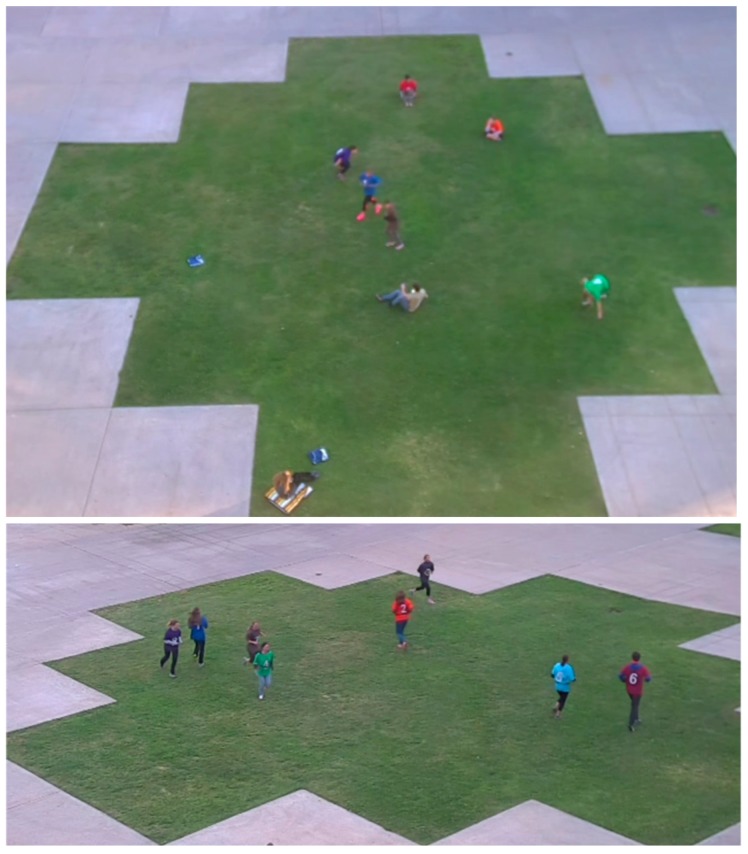
Activity location as viewed from two video recording angles.

Participants were nine healthy adults (age range 19–36 years; 86.7% women) recruited from a convenience sample of employees at the University of California, San Diego. Participants were invited to attend 1–3 approximately 40-min data collection session(s) while wearing an accelerometer and being recorded by three digital video cameras. The accelerometers and video cameras were time-synchronized to the same server clock ≤1 h prior to each data collection session. Each participant also wore a shirt with a distinct color and number on the front and back, to identify when each participant was in and out of the video scene. Shirt colors were not used explicitly in the computer vision processing; they served merely as aids for collection of the ground truth. Each data collection session occurred in the same open activity area on the university campus (see Figure 1) in San Diego, CA, USA during February 2015. Signs were posted to discourage passersby from entering the video scene. The data collection sessions occurred at different times of day, two at 4 p.m. (near sunset) and one at 11 a.m. (midday), so there would be variability in the position of the sun and shadows. This study was approved by the sponsoring institution’s human subjects’ protection committee, and participants provided informed consent.

### 2.2. Activity Session Protocol

A separate activity protocol was developed for each of the three sessions, with each participant being assigned periods of time when they would engage in one of the following activities: sitting, standing, walking, jogging, or out of view of the video cameras. A sample activity session protocol can be found in Appendix A, Table A1. Between one and nine participants were in view of the video cameras throughout each session. At any given time during the session, there were 1–3 different activity blocks occurring simultaneously with 1–4 participants in each block. Each activity block lasted 2–8 min. The number of participants, duration, and activity type for each activity block were randomized, as were the number of different activities occurring at once, each restricted to the aforementioned ranges. If multiple participants were engaged in the same activity block, they were randomized to engage in the activity together (i.e., near each other) or individually (i.e., away from each other). The number of time intervals collected across the 14 different combinations of activity blocks followed a normal distribution (see Table 1).

### 2.3. Measures

#### 2.3.1. Video

Three Foscam FI9826P digital video cameras (resolution = 1280 × 720 pixels) (Foscam, Shenzhen, China) were mounted to a building approximately 30–50 ft high and approximately 65–100 ft from the center of the recording scene. Two cameras faced south at different heights, and one camera faced southwest, all capturing the same activity area. Twenty video frames were recorded from each camera every second.

#### 2.3.2. Accelerometers

Participants wore an ActiGraph GT3X+ accelerometer (aActiGraph LLC, Pensacola, FL, USA) on a belt at their left iliac crest, with vertical axis counts derived for 1-s epochs. Each 1-s epoch was classified as sedentary (0–1.65 counts), light (1.66–32.52 counts), moderate (32.53–95.41 counts), or vigorous (≥95.42 counts) using the well-established Freedson cut points for adults [10]. Metabolic equivalents of task (METs) values were also derived for each 1-s epoch using the equation corresponding to the aforementioned cut points, METs = 1.439008 + 0.000795 × 60 × accelerometer count value. When participants were not in view of the cameras, their accelerometer variables were set to missing. Variables for analyses were derived at the video scene-level, for every second, to represent ecological aggregates across participants. These variables included the number of people (activity volume variables) in the scene, sedentary, light, moderate, vigorous, and moderate-to-vigorous activity (MVPA), total METs across people (total MET-seconds and MET-minutes), proportion (activity distribution variables) of people in each activity category, and average MET-seconds/minutes per person. Data were also aggregated to the minute-level because most end users do not require the granularity of 1-s data, so aggregated data are likely more useful. The same variables were created in the minute-level dataset by taking an average across seconds for the activity volume variables, and dividing by the average number of people in the scene for the activity distribution variables.

### 2.4. Computer Vision Algorithm Development

The final dataset included 6630 s of matched accelerometer and video data per each of the three video recorders (19,890 s of video total). Blocks of 200 s were alternately assigned to a training dataset (*N* = 3830) and testing dataset (*N* = 2800). Features were extracted for each 1-s of video using deep convolutional neural networks [11] for action recognition, learned from a large dataset of human actions [12,13]. Four classification models (one for each activity category) were used to predict the number of participants in each activity category and trained end-to-end with stochastic gradient descent [14]. For predicting total MET-seconds in the scene (across people), a recurrent neural network called Long Short-Term Memory [15] was used to connect temporal features. MET-seconds predictions were made using a regression approach trained with root mean square propagation [16].

### 2.5. Analyses

Agreement between accelerometer/criterion and camera/predicted measurements for each variable was assessed using intraclass correlation coefficients (ICCs), mean differences (camera/ predicted value minus accelerometer/criterion value; referred to as bias), and standard deviations (SD) of the mean differences. Second-level data and minute-level data were analyzed separately. Bias indicates whether the computer vision algorithm tended to over- or under-estimate each variable (accuracy). The SD of the bias (biasSD) captures the variance in the bias and indicates the limits of agreement, which can be used to infer individual-level validity (individual here refers to time intervals rather than participants). Percent bias (%bias) was calculated as bias ÷ mean value from the accelerometer, and %biasSD was calculated as biasSD ÷ mean value from the accelerometer. Our purpose was to assess each camera’s agreement with the accelerometer, and toward this end we computed the ICC (ICC2) [17], bias, and biasSD for each of the three cameras individually. This approach allows variability across cameras/positions on the three measures to be evaluated, giving a sense of the variability in agreement one might expect across cameras/positions in real-life application.

To assess overall agreement between predicted and criterion measurements for the minute-level variables, we combined the data from the three cameras and plotted the predicted and criterion measurements against each other in a single plot for each variable. The line of perfect agreement (Y = X) was displayed in each plot, allowing visual assessment of the overall agreement and consistency of the bias across low and high values for each variable. Values of Lin’s concordance correlation coefficient (CCC) [18] were also calculated using the combined data for each variable to quantify the overall agreement between predicted and criterion values. Lin’s CCC measures the goodness of fit around the line of perfect agreement and while it can yield values very close to the ICC, it accounts for systematic bias.

Thresholds for interpreting bias and biasSD vary by the inferences that will be drawn from the test, and there is no accepted standard for ecological physical activity measures. For the present study, we considered a bias of ≤20% to be acceptable for sample-level validity, and a %bias ± 2 × %biasSD (i.e., limits of agreement) of ≤30% to be acceptable for individual-level validity. Criteria for interpreting ICCs and CCCs were: poor (≤0.40), fair (0.41–0.60), good (0.61–0.80), and excellent (0.81–1.0) [19]. Statistical analyses were performed in R.

## 3. Results

The characteristics of the dataset with regards to the distribution of people densities and activity intensities are presented in Table 2. The dataset was approximately evenly split between more (5–9 people) and less (1–4 people) people-dense scenes. Sedentary, moderate, and vigorous activities were each represented in over half of the scenes, whereas light activity was represented in only 17.4% of the scenes.

### 3.1. Minute-Level Validity

Table 3 shows results of agreement analyses for the minute-level dataset. The directions of the biases indicated that on average the algorithm overestimated people in vigorous activity by 0.22–0.32 people (19–27.6%) (0.04–0.06 for proportion of people (17.4–26.1%)) and underestimated people in moderate activity by 0.09–0.12 people (8.3–11.1%) (0.03–0.04 for proportion of people (9.7–12.9%)) and in light activity by 0.06–0.08 people (30–40%) (0.02 for proportion of people (33.3%)). The algorithm underestimated total MET-minutes on average by 1.68–3.07 MET-minutes (9.3–17.1%) and average MET-minutes per person by 0.19–0.34 MET-minutes (4.7–8.3%) for two cameras (0.04 MET-minutes (1%) overestimation by the other camera).

Although the mean bias estimates were favorable for several of the variables investigated, the SDs and limits of agreement around the bias estimates were moderate-to-large relative to the mean. For the number and proportion of people sedentary and participating in MVPA, %biasSDs ranged from 19.5% to 32.4%. For total MET-minutes in the scene and average MET-minutes per person, %biasSDs ranged from 38.7% to 49.4%. No limits of agreement (%bias ± 2 × %biasSD) were within 30% of the criterion mean. For example, the lower and upper limits for the proportion of people engaged in MVPA were −37.5% and 47% (averaged across cameras), and these limits of agreement were among the smallest/narrowest of the variables investigated.

ICCs were excellent (≥0.82) for the number and proportion sedentary, moderate, vigorous, and engaged in MVPA, and poor (0.14–0.36) for the number and proportion of people in light activity. ICCs were good for total MET-minutes (0.68–0.79) and fair (0.43–0.59) for average MET-minutes per person.

Bias estimates did not vary across cameras by more than 0.11 people (10%) (0.02 for proportions (8.7%)) for each activity category, or by more than 1.4 for total MET-minutes (7.8%) and 0.38 for average MET-minutes per person (9.3%). %biasSDs did not vary across cameras by more than 0.28 people (21.6%) (0.04 for proportion (16.7%)) for each activity category, or by more than 1.2 for total MET-minutes (6.7%) and 0.27 for average MET-minutes per person (6.6%). ICCs did not vary across cameras by more than 0.07 for all metrics except total MET-minutes and average MET-minutes per person, which varied by up to 0.16.

**Figure 2:**
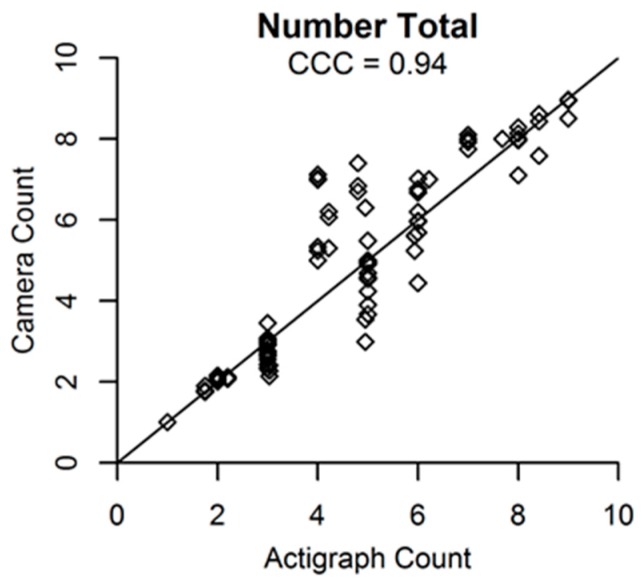
Agreement plots for the total number of participants in the scene (*N* = 144 predictions from 48 min).

**Figure 3:**
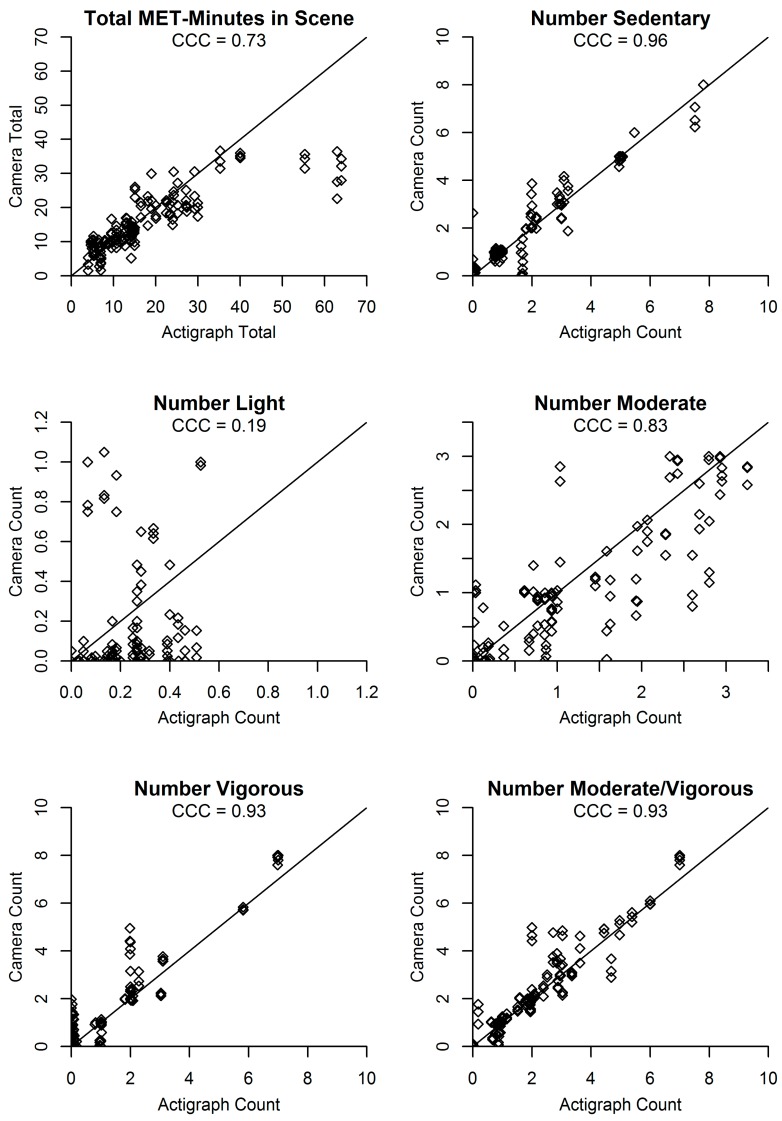
Agreement plots for total MET-minutes and number of participants in each activity category (*N* = 144 predictions from 48 min).

**Figure 4:**
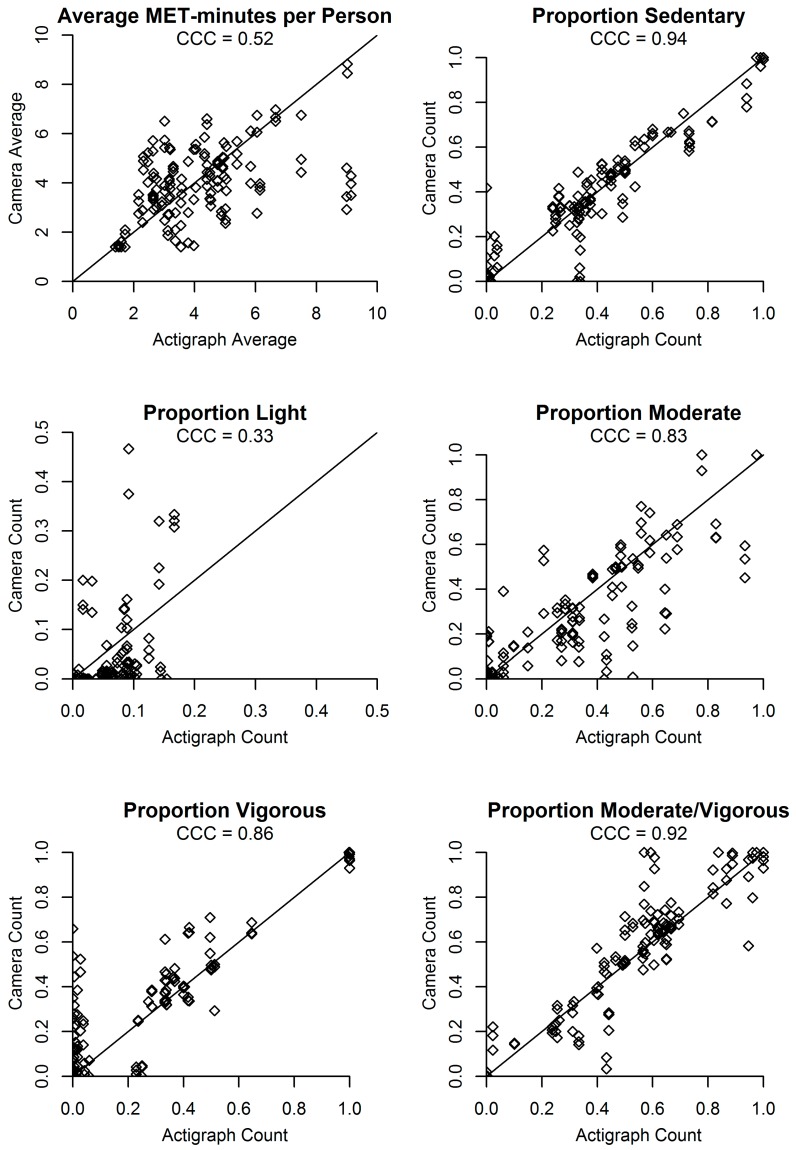
Agreement plots for average MET-minutes per person and proportion of participants in each activity category (*N* = 144 predictions from 48 min).

Figure 2, Figure 3 and Figure 4 present agreement plots of the criterion against predicted values for each variable in the minute-level dataset. CCCs were excellent for the number and proportion of people in each activity category, except for light activity which had poor CCCs. The CCC for total MET-minutes was good and for average MET-minutes per person (which takes into account the predicted number of people in the scene) was fair. In each plot, bias appeared to be relatively consistent across low and high values of the accelerometer/criterion, with the exception of total MET-minutes in the scene and average MET-minutes per person, which showed greater underestimation as the criterion values for total MET-minutes and average MET-minutes per person increased.

### 3.2. Second-Level Validity

Results for the second-level analyses were similar to the minute-level analyses with regard to the bias estimates (see Appendix A, Table A2). However, the second-level biasSDs and ICCs and were generally less favorable than the minute-level biasSDs and ICCs, as one would expect given the reduction in variance gained from averaging the seconds to minutes.

## 4. Discussion

Present findings show a proof of concept for using computer vision for continuous ecological assessment of both volume and distribution of physical activity in open spaces. Sample-level validity estimates were favorable (bias ≤ 20% and good to excellent ICCs) for 1-s and 1-min estimates of the number and proportion of sedentary people and those participating in MVPA, and total MET-minutes in the scene. The number and proportion of sedentary participants and those participating in MVPA had the strongest validity, which is a positive finding because these metrics are among the most commonly used variables from direct observation measures [5,6,7]. Total MET-minutes, another important metric, can be used to show the total amount of physical activity conducted in a given setting. The present research, and one previous study [8], suggest that automated ecological assessment of physical activity using computer vision has promise for providing continuous monitoring/feedback and evaluating interventions in settings, such as parks and schoolyards. Before such systems are ready for application, more research is needed to test the computer vision algorithms in a larger number of settings, with larger groups, and a broader range of activities in naturalistic conditions.

The number and proportion of people sedentary and in MVPA had the strongest validity of the variables tested, based on agreement and linear associations with the criterion values. The algorithms overestimated vigorous activity and underestimated moderate and light activity, but combining moderate and vigorous activity improved validity estimates, which is a positive finding given that MVPA is the most commonly-derived activity metric in public health studies. The algorithm had acceptable/good validity for predicting total MET-seconds/minutes in the scene, but only moderate validity for average MET-seconds/minutes per person. The poorer performance for average MET-seconds/minutes per person was due to the number of people in the scene (denominator) being overestimated and total MET-seconds/minutes (numerator) being underestimated. Predicting MET-seconds/minutes is a more difficult task than predicting activity categories because more precision is required, but total MVPA-minutes (the number of people in MVPA × the minutes observed) can also provide a meaningful measure of physical activity volume. Only minimal differences in validity estimates were observed across the three cameras, suggesting that multiple camera locations, heights, and angles may be feasible for use, though the lowest height used in the present study was 30 ft.

The limits of agreement (%bias ± 2 × %biasSD) were outside of the desired range for the individual level (i.e., for individual minutes or seconds) validity for all variables (i.e., not within 30% of the criterion mean). This means there was a relatively wide variation in bias across time intervals, so although total MET-minutes, for example, can be predicted accurately on average, predictions may have less than desirable accuracy for any given minute or second. BiasSDs were substantially better in the minute-level vs. second-level dataset, and would likely continue to improve with larger levels of aggregation (e.g., 5 min), which are more likely to be desired by end users. It is possible that the %biasSDs would be more favorable in scenes with a larger number of people. In environments with only a handful of people, such as in the present study, a misclassification of one or two people’s activity levels could lead to a >50% misclassification in the number of people participating in MVPA and total MET-minutes, whereas misclassifying one or two people in an environment with 30–40 people would have relatively small impacts on validity and bias estimates. Thus, future studies should include a larger number and more variability in the number of people, record for a longer duration to support aggregating longer time intervals, and identify predictors of bias to inform when the data should and should not be used in inference-making.

The activity volume and distribution variables derived from the computer vision algorithms investigated in this study parallel those derived by commonly-used direct observation tools, such as SOPARC and SOFIT [3,4]. These variables can be used for many purposes, such as estimating the contribution of a park or schoolyard to population health, comparing use and activity across settings (e.g., across parks or areas within a park) and time (e.g., day of week or time of year), and comparing activity across groups or interventions (e.g., comparing PE classes) [5,6,7]. Automated assessment would be advantageous in situations where multiple or ongoing assessments are desired, but manpower resources are limited. Ongoing automated assessment could be used to provide ongoing feedback to support data-driven adaptive interventions [20,21,22,23] (e.g., programming or environmental), which could significantly improve the health impacts of parks and schools. Although the use of video cameras in parks and schoolyards is controversial, embedded cameras (e.g., for security purposes) are becoming more ubiquitous in these settings, and this trend will likely continue [24,25,26,27,28]. Thus, leveraging this information for public health should be a priority. However, a substantial amount of work is needed to identify strategies for developing, installing, and using embedded camera systems for public health research, as many considerations exist, such as hardware development and management, control and transfer of data, ethics, and feedback of information to end users. Costs associated with embedded camera systems would likely include camera/hardware purchase and installation, power supply, data transfer and storage, software purchase, and hardware and software maintenance. However, it is currently unclear how these costs would compare to costs of conducting SOPARC assessments, which require substantial ongoing resources for training, labor, supervision, and data reduction and processing. Many challenges of automated systems are also relevant to direct observation measures, such as weather constraints and movement of people across areas/zones of the park or schoolyard. Some challenges may be unique to automated systems and should be further investigated, such as confusion between animals and people and greater misclassification in shadowy/dark and occluded areas.

### Strengths, Limitations, and Future Directions

This study was among the first to develop and test computer vision algorithms for ecological assessment of physical activity in outdoor settings. The use of accelerometers for training and testing the algorithms can be considered a strength because of the high level of accuracy for assessing activity intensity and the high frequency of data provided (i.e., data every second). SOPARC was not used as a comparison measure because SOPARC observations are traditionally collected infrequently (e.g., 15 min) across multiple settings and observation days, and the present sample for testing the algorithms was small (one setting, three observation days, 48 min in total). An important research question is to identify whether computer vision algorithms are more or less accurate than SOPARC as compared to accelerometry, so this should be investigated in future studies with larger sample sizes. Another strength was the balance of activity combinations across sitting, standing, walking, and jogging, although future studies should investigate more real-world activities because the prescribed nature of the activities in the present study may limit generalizability to other activities. Other limitations included the use of only one setting, a relatively small number of participants, and no obstructions, all of which limit generalizability to other settings. The next steps should be to test the computer vision algorithms in more diverse settings such as those with larger numbers of people, different age groups, obstructions/occlusions (e.g., trees and playground facilities), variability in lighting (e.g., due to cloud cover), and different surfaces (e.g., grass vs. pavement). We did not investigate whether computer vision could be used to identify gender, race/ethnicity, or age group, which are often captured in direct observation measures, and this capability should be investigated in the future [3,4].

## 5. Conclusions

Computer vision appears promising for automated ecological assessment of physical activity in open outdoor settings, but further work is needed to create generalizable algorithms. The computer vision algorithms tested in the present study had strong sample-level validity for ecological assessment of sedentary activity and MVPA, and moderate sample-level validity for total MET-minutes. Although the algorithms were designed to adapt to different settings with variations in lighting, backgrounds, and occlusions, further development and testing is needed in a diverse range of parks/schoolyards and activities before such systems are ready for application. Automated ongoing assessment and feedback would provide several novel advantages over direct observation tools. If automated assessment of physical activity was integrated into management processes, it would have great potential for improving the impact of parks and schools on population health.

## Figures and Tables

**Table 1:** Distribution of activity block combinations in the full dataset.

Activity Block Combinations	Number of Simultaneous Activities	Number of Seconds of Data
Sitting only	1	420
Standing only	1	540
Walking only	1	840
Jogging only	1	720
Sitting and standing	2	720
Sitting and walking	2	720
Sitting and jogging	2	780
Standing and walking	2	660
Standing and jogging	2	480
Walking and jogging	2	420
Sitting, standing, and walking	3	240
Sitting, standing, and jogging	3	240
Sitting, walking, and jogging	3	240
Standing, walking, and jogging	3	180
Total	-	7200 ^a^

^a^ The final dataset included 6630 s of video recording due to missing video data.

**Table 2:** Characteristics of testing dataset (*N* = 2800 s).

Characteristics	Number (%)
Seconds with 1–4 people in scene	1322 (47.2%)
Seconds with 5–9 people in scene	1478 (52.8%)
Seconds with ≥1 person sedentary	2082 (74.4%)
Seconds with ≥1 person in light activity	488 (17.4%)
Seconds with ≥1 person in moderate activity	1594 (56.9%)
Seconds with ≥1 person in vigorous activity	1509 (53.9%)
Seconds with ≥1 person in MVPA	2454 (87.6%)
Seconds with <3 MET-seconds per person	1358 (48.5%)
Seconds with ≥3 MET-seconds per person	1442 (51.5%)

MET = metabolic equivalents of tasks; MVPA = moderate to vigorous physical activity.

**Table 3:** Agreement and associations between the video-based algorithm (test measure) and accelerometry (criterion measure) in the aggregated minute-level dataset (*N* = 48 min per camera).

Variables	Criterion	Camera 1			Camera 2			Camera 3		
	Mean (SD)	Bias (SD)	%Bias (SD)	ICC	Bias (SD)	%Bias (SD)	ICC	Bias (SD)	%Bias (SD)	ICC
Activity volume variables										
Number people in scene	4.34 (2.09)	0.15 (0.74)	3.5% (17.1)	0.94	0.08 (0.79)	1.8% (18.2)	0.93	0.18 (0.77)	4.1% (17.7)	0.94
Number of people sedentary	1.90 (1.89)	0 (0.37)	0% (19.5)	0.98	0.01 (0.65)	0.5% (34.2)	0.94	0.11 (0.51)	5.8% (26.8)	0.96
Number of people light	0.20 (0.16)	−0.06 (0.27)	−30.0% (135.0)	0.24	−0.08 (0.29)	−40.0% (145.0)	0.14	−0.07 (0.27)	−35.0% (135.0)	0.21
Number of people moderate	1.08 (1.02)	−0.12 (0.58)	−11.1% (53.7)	0.83	−0.12 (0.52)	−11.1% (48.1)	0.85	−0.09 (0.59)	−8.3% (54.6)	0.83
Number of people vigorous	1.16 (1.70)	0.32 (0.77)	27.6% (66.4)	0.90	0.28 (0.59)	24.1% (50.9)	0.93	0.22 (0.52)	19.0% (44.8)	0.95
Number of people in MVPA	2.24 (1.73)	0.20 (0.74)	8.9% (33.0)	0.91	0.15 (0.57)	6.7% (25.4)	0.95	0.13 (0.61)	5.8% (27.2)	0.94
Total MET-minutes in scene	18.0 (14.1)	−1.8 (7.7)	−10.0% (42.8)	0.79	−3.1 (8.9)	−17.1% (49.4)	0.68	−1.7 (8.5)	−9.3% (47.2)	0.72
Activity distribution variables										
Proportion of people sedentary	0.40 (0.28)	−0.01 (0.08)	−2.5% (20.0)	0.96	0 (0.12)	0% (30.0)	0.91	0.01 (0.08)	2.5% (20.0)	0.96
Proportion of people light	0.06 (0.05)	−0.02 (0.08)	−33.3% (133.3)	0.33	−0.02 (0.07)	−33.3% (116.7)	0.32	−0.02 (0.08)	−33.3% (133.3)	0.36
Proportion of people moderate	0.31 (0.28)	−0.03 (0.15)	−9.7% (48.4)	0.85	−0.04 (0.16)	−12.9% (51.6)	0.82	−0.03 (0.15)	−9.7% (48.4)	0.84
Proportion of people vigorous	0.23 (0.28)	0.06 (0.14)	26.1% (60.9)	0.86	0.06 (0.14)	26.1% (60.9)	0.85	0.04 (0.13)	17.4% (56.5)	0.88
Proportion of people MVPA	0.54 (0.28)	0.02 (0.11)	3.7% (20.4)	0.93	0.03 (0.13)	5.6% (24.1)	0.90	0.01 (0.11)	1.9% (20.4)	0.93
Average MET-minutes per person	4.08 (1.92)	−0.19 (1.58)	−4.7% (38.7)	0.59	−0.34 (1.85)	−8.3% (45.3)	0.43	0.04 (1.69)	1.0% (41.4)	0.56

Bias = predicted (camera) − criterion (accelerometer). %Bias = bias ÷ criterion (accelerometer). ICC = intraclass correlation coefficient. MET = metabolic equivalents of tasks. MVPA = moderate to vigorous physical activity. SD = standard deviation.

## Appendix A

**Table A1:** Sample activity session protocol.

Minute	Combination Number ^a^	Total Number of People	Blocks		
			Group A	Group B	Group C
0	1	3		Three people sitting individually	
1	1	3			
2	1	3			
3	1	3			
4	1	3			
5	1	3			
6	1	3			
7	2	2			Two people standing together
8	2	2			
9	2	2			
10	3	1		One person walking	
11	3	1			
12	3	1			
13	10	5	Two people jogging individually		Two people walking together
14	10	5			
15	10	5			
16	14	5			Two people standing individually
17	9	6		Two people standing individually	
18	9	6			
19	9	6			
20	5	8	Four people sitting individually		
21	5	8			
22	5	8			
23	5	8			Two people sitting individually
24	7	8		Two people jogging together	
25	7	8			
26	9	3			One person standing
27	9	3			
28	9	3			
29	8	3		Two people walking together	
30	8	3			
31	8	3			
32	8	3			
33	8	3			
34	8	7	Three people sitting individually		Two people standing together
35	5	5			
36	7	4			One person jogging
37	7	4			
38	6	4			One person walking
39	6	4			

^a^ The activities were sitting, standing, walking, and jogging; 1–3 activities were engaged in simultaneously (by different groups of participants), resulting in 14 possible combinations of activities across participants.

**Table A2:** Agreement and associations between the video-based algorithm (test measure) and accelerometry (criterion measure) in the unaggregated second-level dataset (*N* = 2800 seconds per camera).

Variables	Criterion	Camera 1			Camera 2			Camera 3		
	Mean (SD)	Bias (SD)	%Bias (SD)	ICC	Bias (SD)	%Bias (SD)	ICC	Bias (SD)	%Bias (SD)	ICC
Activity volume variables										
Number people in scene	4.47 (2.16)	0.18 (0.94)	4.0% (21.0)	0.91	0.13 (1.16)	2.9% (26.0)	0.87	0.21 (1.00)	4.7% (22.4)	0.9
Number of people sedentary	1.95 (1.97)	0.01 (0.65)	0.5% (33.3)	0.95	0.04 (1.06)	2.1% (54.4)	0.86	0.13 (0.86)	6.7% (44.1)	0.91
Number of people light	0.20 (0.45)	−0.05 (0.55)	−25.0% (275.0)	0.1	−0.07 (0.54)	−35.0% (270.0)	0.07	−0.06 (0.54)	−30.0% (270.0)	0.12
Number of people moderate	1.01 (1.11)	−0.11 (0.93)	−10.9% (92.1)	0.64	−0.12 (0.95)	−11.9% (94.1)	0.63	−0.07 (0.95)	−6.9% (94.1)	0.63
Number of people vigorous	1.30 (1.84)	0.33 (1.08)	25.4% (83.1)	0.85	0.28 (1.09)	21.5% (83.8)	0.84	0.22 (1.06)	16.9% (81.5)	0.86
Number of people in MVPA	2.32 (1.87)	0.22 (1.03)	9.5% (44.4)	0.86	0.16 (1.04)	6.9% (44.8)	0.86	0.15 (0.98)	6.5% (42.2)	0.88
Total MET-minutes in scene	19.1 (15.1)	−3.1 (10.2)	−16.0% (53.4)	0.67	−4.2 (11.1)	−21.7% (58.1)	0.56	−2.88 (11.0)	−15.1% (57.7)	0.6
Activity distribution variables										
Proportion of people sedentary	0.40 (0.30)	−0.01 (0.14)	−2.5% (35.0)	0.89	−0.01 (0.20)	−2.5% (50.0)	0.79	0 (0.16)	0% (40.0)	0.87
Proportion of people light	0.05 (0.13)	−0.01 (0.17)	−20.0% (340.0)	0.11	−0.02 (0.15)	−40.0% (300.0)	0.13	−0.01 (0.16)	−20.0% (320.0)	0.13
Proportion of people moderate	0.29 (0.31)	−0.03 (0.25)	−10.3% (86.2)	0.66	−0.03 (0.27)	−10.3% (93.1)	0.64	−0.03 (0.26)	−10.3% (89.7)	0.64
Proportion of people vigorous	0.26 (0.29)	0.05 (0.21)	19.2% (80.8)	0.76	0.06 (0.22)	23.1% (84.6)	0.72	0.04 (0.22)	15.4% (84.6)	0.75
Proportion of people MVPA	0.54 (0.31)	0.03 (0.19)	5.6% (35.2)	0.81	0.03 (0.22)	5.6% (40.7)	0.76	0.01 (0.20)	1.9% (37.0)	0.8
Average MET-minutes per person	4.20 (2.05)	−0.30 (2.12)	−7.1% (50.5)	0.46	−0.35 (2.52)	−8.3% (60.0)	0.31	−0.09 (2.31)	−2.1% (55.0)	0.4

Bias = predicted (camera) − criterion (accelerometer). %Bias = bias ÷ criterion (accelerometer). ICC = intraclass correlation coefficient. METs = metabolic equivalents of tasks. MVPA = moderate to vigorous physical activity. SD = standard deviation.
